# Role of urinary H_2_O_2_, 8-iso-PGF_2_α, and serum oxLDL/β2GP1 complex in the diabetic kidney disease

**DOI:** 10.1371/journal.pone.0263113

**Published:** 2022-04-05

**Authors:** Rani Sauriasari, Afina Irsyania Zulfa, Andisyah Putri Sekar, Nuriza Ulul Azmi, Xian Wen Tan, Eiji Matsuura

**Affiliations:** 1 Faculty of Pharmacy, Universitas Indonesia, Depok, Indonesia; 2 Department of Cell Chemistry, Okayama University Graduate School of Medicine, Dentistry, and Pharmaceutical Sciences, Okayama, Japan; 3 Collaborative Research Center (OMIC), Okayama University Graduate School of Medicine, Dentistry, and Pharmaceutical Sciences, Okayama, Japan; 4 Neutron Therapy Research Center, Okayama University, Okayama, Japan; University Medical Center Utrecht, NETHERLANDS

## Abstract

Oxidant species is reported as a major determinant in the pathophysiology of diabetic kidney disease. However, reactive oxygen species (ROS) formation in the initial phase and progressing phase of diabetic kidney disease remains unclear. Therefore, we conducted this study to find out what ROS and their modified product are associated with eGFR in type 2 diabetes mellitus (T2DM) patients. A cross-sectional study was performed on 227 T2DM patients. The study subjects were divided into three groups based on their eGFR stage (Group 1, eGFR > 89 ml/min/1.73 m^2^; Group 2, eGFR = 60–89 ml/min/1.73 m^2^; and Group 3, eGFR < 60 ml/min/1.73 m^2^). Enzyme-linked immunosorbent assay (ELISA) was used to measure serum oxLDL/β_2_GPI complex and urinary 8-iso-PGF2α, while ferrous ion oxidation xylenol orange method 1 (FOX-1) was used to measure urinary hydrogen peroxide (H_2_O_2_). H_2_O_2_ significantly decreased across the groups, whereas OxLDL/β_2_GPI complex increased, but not significant, and there was no trend for 8-iso-PGF2α. Consistently, in the total study population, only H_2_O_2_ showed correlation with eGFR (r = 0.161, p = 0.015). Multiple linear regression analysis showed that significant factors for increased eGFR were H_2_O_2_, diastolic blood pressure, and female. Whereas increased systolic blood pressure and age were significant factors affecting the decrease of eGFR. We also found that urinary H_2_O_2_ had correlation with serum oxLDL/β_2_GPI complex in total population. This finding could lead to further research on urinary H_2_O_2_ for early detection and research on novel therapies of diabetic kidney disease.

## Introduction

Diabetes mellitus is one of the most common non-communicable diseases. According to the International Diabetes Federation (IDF) Atlas 9^th^ edition, in 2019 463 million people were living with diabetes and about three in four of those people (352 million) were of working age (i.e. 20–64 years old). This number is expected to increase to 417 million by 2030 and 486 million by 2045. In 2019, Indonesia was ranked seventh in the world for the highest prevalence of diabetes in the world and is predicted to remain so until 2030 [1].

Diabetes mellitus is associated with a high incidence of atherosclerotic complications that result from chronic metabolic abnormalities such as hyperglycemia and hyperlipidemia, in type 2 diabetes mellitus (T2DM) may enhance systemic oxidative stress, resulting in the excessive production of lipid peroxides and subsequently contributing to the pathogenesis of atherothrombosis and microangiopathy, which is the common pathogenetic mechanism underlying diabetic vascular complications [2–4]. Diabetic nephropathy is one of the chronic complications in the form of decreased renal function characterized by proteinuria, hypertension, and the declining glomerular filtration rate (GFR) [5].

Continuous hyperglycemia and hyperlipidemia induce overproduction of reactive oxygen species (ROS), resulting in renal cell apoptosis, which plays an important role in the molecular mechanism of renal damage in diabetes [6, 7]. Peroxides, free radicals, protein-, lipid- and DNA-modified substances are oxidative stress-related markers that have a pivotal role in cell damage [7, 8]. Hydrogen peroxide (H_2_O_2_) is one of a few peroxides used as a biomarker of oxidative stress [9, 10]. The unique characteristic of H_2_O_2_ is its ability to diffuse into the cell, forming hydroxyl radicals, and has the potential to attack biological molecules such as DNA, proteins, and lipids leading to cell damage [11, 12].

Lipid peroxidation has been considered to be a key mechanism for the development of atherosclerosis and vascular damage [7]. 8-iso-PGF2α are prostaglandin-like compounds produced from the oxidation of arachidonic acid through non-enzymatic processes that are catalyzed by free radicals such as H_2_O_2_. The 8-iso-PGF2α production will circulate in blood vessels and be excreted in the urine of humans and animals [13, 14]. 8-iso-PGF2α is also capable of activating platelets through its ability to bind to thromboxane prostanoid receptors [15].

Another oxidatively modified-lipid product is the OxLDL/β_2_GP1 complex. OxLDL/β_2_GP1 complex is an oxidized product of LDL cholesterol in which some studies showed that OxLDL binds to endogenous β_2_GPI to form oxLDL/β_2_GP1 complex that can be found in the bloodstream of patients with chronic renal disease and diabetes mellitus [16]. The oxLDL/β_2_GP1 complex represents an important pathogenic event in the progression of atherosclerotic lesions and can be used to represent a substitute marker for oxidative inflammation in diabetes mellitus [17]. In chronic renal disease, an increase in oxidized LDL (OxLDL) participates in the development of glomerulosclerosis and interstitial fibrosis [18, 19].

Although oxidative stress is known to contribute to diabetic kidney disease, it is still unknown whether oxidative stress is associated with renal hyperfiltration in the initial phase (G1 stage) of chronic kidney disease or associated with renal hypofiltration in the progressing phase (G3a-G5 stage). The purpose of this study was to determine whether oxidative stress markers such as H_2_O_2_, 8-iso-PGF2α, and oxLDL/β_2_GP1 complex affected renal function as shown by eGFR level in T2DM patients. In this study, we used the CKD-EPI equation to determine the patient’s eGFR level, since the CKD-EPI equation shows less bias, greater accuracy, more precision, and also commonly used in clinical practice settings [20].

## Materials and methods

### Study design and study population

A multicenter study was conducted at RSK Sitanala Tangerang and Community Health Center, Pasar Minggu District, South Jakarta, Indonesia during 2015, 2016, and 2019. The study was approved by the Ethical Committee, Faculty of Medicine Universitas Indonesia of Cipto Mangunkusumo Hospital (No.76/UN2.F1/ETIK/2015 and 0222/UN2.F1/ETIK/2018) and RSK Sitanala Ethical Committee (No. DM.04.04/1101663/2015). Written informed consent was obtained from all subjects. This study was performed on 227 T2DM patients who were divided based on their eGFR value. Group 1 included patients in G1 stage (eGFR > 89 mL/min/1.73 m^2^) (n = 121); Group 2 included patients in G2 stage (eGFR 60–89 ml/min/1.73 m^2^) (n = 74), and Group 3 included patients in G3a to G5 stages (eGFR < 60 ml/min/1.73 m^2^) (n = 32). Inclusion criteria in this study were T2DM-diagnosed patients aged ≥ 36 years (late adulthood, according to age classification by Ministry of Health, Republic of Indonesia), fasting for at least eight hours before sampling, and willingness to give informed consent. The exclusion criteria were patients who could not provide blood and/or urine specimens or did not have complete clinical data; pregnant and breastfeeding women; patients with severe anemia and/or those receiving blood transfusions; patients suffering from heart disease, stroke, impaired liver function, and infectious disease (e.g. tuberculosis); and patients in kidney failure and/or currently undergoing renal replacement therapy.

### Materials and apparatus

This study used the following apparatus: microplate reader (SUNRISE; Serial number: 1001006648; Firmware: V 3.32 08/07/08; XFLUOR4 Version: V 4.51); Multi-mode Microplate Reader FlexStation^®^ (Molecular Devices, USA); plate shaker (Heidolph Unimax 1010); microcentrifuge (Allegra 64 R); Afinion^TM^ AS100 Analyzer (Abbott); and ultrafreezer -80°C (Biomedical, Lab Tech). Chemicals used in this study were creatinine detection kit (Enzo Life Sciences, Cat. No. ADI 907–030 A); 8-iso-PGF2α ELISA kit (Enzo Life Sciences, Cat. No. ADI-900-010); AtherOx^®^ (oxLDL/β_2_GPI antigen complexes) kit (Corgenix, USA); and H_2_O_2_ reagents (xylenol orange, sorbitol, catalase enzyme, 30% H_2_O_2_, sulfonic acid, Fe(NH_4_)_2_(SO_4_)6H_2_O, NaH_2_PO_4_.H_2_O, Na_2_HPO_4_2H_2_O, and deionized water).

### Collection of urine and serum sample

First morning urine samples (30 mL) were collected from each subject. Samples were stored at -80°C until analysis. Urine samples were divided into 3–5 aliquots and were used for the determination of urinary creatinine, albumin, H_2_O_2_, and 8-iso-PGF2α. Blood samples were collected from each participant through the vena mediana cubiti into vacutainer tubes. The blood was centrifuged at 1500–2000 rpm for 10 minutes, separated and stored at -80°C until required for analysis. The sera of the blood samples were used for the determination of oxLDL/β_2_GP1 complex and serum creatinine.

### Measurement of serum creatinine

Blood samples (10 mL) were taken from the subjects by certified phlebotomists from Prodia laboratory, an accredited clinical laboratory. Measurement of serum creatinine level in the sample was carried out by Prodia Laboratory by the enzymatic colorimetry method using creatininase, creatinase, and sarcosinase [21].

### Measurement of eGFR

Based on the results of serum creatinine measurements, the eGFR values were calculated using the CKD-EPI equation [22].

### Measurement of UACR

UACR was measured using Afinion^TM^ AS100 analyzer. The assay analyzes both the albumin and creatinine in a spot urine sample simultaneously within a single device. Albumin is quantified using a immunometric membrane flow through assay, using monoclonal antibody-coated membrane and monoclonal antibodies conjugated to colloid gold. Creatinine is quantified using an enzymatic colorimetris tests involving 4 enzymatic steps [23].

### Measurement of serum OxLDL/β2GPI complex

Serum oxLDL/β_2_GPI complex were measured by AtherOx ELISA kits (Corgenix, USA). The test was performed using a sandwich ELISA method. Diluted serum samples, calibrators, and controls were incubated in microwells coated with purified anti-human monoclonal antibody directed only to complexed β_2_GPI. A 100 μL of 1:50 dilution serum samples were incubated in microwells at room temperature for one hour. The microwells were washed four times with phosphate-buffered saline containing 0.05% polysorbate-20. After washing, anti-human apoB100 (LDL) monoclonal antibody conjugated to biotin was added to form complexes with the bound antigen, followed by 30 minutes’ incubation at room temperature. Following further washing, a horseradish peroxidase-conjugated streptavidin (HRP-SA) was added to form complex with the bound biotin-conjugated antibody and incubated for 30 minutes. Following further washing, the bound HRP-SA conjugate was assayed by the addition of tetramethylbenzidine (TMB) and H_2_O_2_ chromogenic substrate and incubated for 30 minutes. The color developed in the wells was directly proportional to the serum concentration of OxLDL/β_2_GPI antigen complex. The reaction was stopped with 0.36 N sulfuric acid. The optical density was read at a 450 nm (650 nm as reference).

### Measurement of urinary 8-iso-PGF_2_α

The analysis of 8-iso-PGF2α was carried out using competitive ELISA with commercial immunoassay kits for 8-iso-PGF2α (catalog #ADI-900-010, Enzo Life Sciences, Farmingdale, NY, USA). The kit used a polyclonal antibody to bind to 8-iso-PGF2α in a competitive manner, with 8-iso-PGF2α in a sample or with an alkaline phosphatase molecule which has 8-iso-PGF2α covalently attached to it. After simultaneous incubation at room temperature, the excess reagents were washed away and substrate was added. After a short incubation time, the enzyme reaction stopped and the yellow color generated was read on a microplate reader at 405 nm. The intensity of yellow color was inversely proportional to the concentration of 8-iso-PGF2α in either the standards or the samples. The measured optical density was used to calculate the concentration of 8-iso-PGF2_α_.

### Measurement of urinary H_2_O_2_

Urinary H_2_O_2_ measurement was conducted by ferrous ion oxidation xylenol orange method 1 (FOX-1) assay [24]. This method is based on the oxidation of the Fe^2+^ reagent to Fe^3+^ by an oxidizing agent (H_2_O_2_). The oxidized Fe^3+^ will bind to ethylene xylenol orange (XO), generating a color complex that has maximum absorption at 560 nm. Then, 20 μL of the urine samples were incubated with 20 μL of catalase solution (2200 U/ml in 25 mM phosphate buffer, pH 7.0). The samples were reacted with 160 μL of FOX-1 reagent pH 1.7–1.8 by addition of Na_2_HPO_4_ at room temperature for 30 minutes. Absorbance was measured with a microplate reader at 560 nm. Urinary H_2_O_2_ concentration was determined by calculating the absorbance difference in the samples with and without catalase [11].

### Statistical analysis

Comparison among groups was carried out using the chi-square test for categorical variables. Kruskall–Wallis test followed by Mann–Whitney test were used to analyze nonparametric data, and one-way ANOVA testing was used for parametric data. Correlation between variables was analyzed using Spearman or Pearson tests, depended on data normality. A *p*-value of less than 0.05 was considered statistically significant. Multivariate analysis was also used to find the patterns and relationships between multiple variables, enabling factors affecting the results to be identified. All analysis was performed using IBM SPSS Statistics 22 software.

## Results and discussion

### Characteristics of the study groups

Data were obtained from 227 subjects (47 men and 180 women) consisted of 121 subjects with eGFR > 89 mL/min/1.73 m^2^, 74 subjects with eGFR 60–89 mL/min/1.73 m^2^, and 32 subjects with eGFR < 60 mL/min/1.73 m^2^ (Table 1). There were no significant differences in the proportion of gender, weight, height, body mass index (BMI), exercise habit, smoking habit, blood pressure, HbA1c, urine creatinine, and albuminuria status in the three groups of study subjects (Table 1). All groups mainly consisted of female subjects. The mean age of the study subjects in all groups were above 55 years and subjects in group 1 were younger than group 2 and group 3 (*p* < 0.001).

**Table 1:** Basic characteristics of study subjects.

Characteristic of study subjects	Group 1	Group 2	Group 3	*p*
	eGFR > 89 mL/min/1.73 m^2^ (n = 121)	eGFR 60–89 mL/min/1.73 m^2^ (n = 74)	eGFR < 60 mL/min/1.73 m^2^ (n = 32)	
	Mean (%) or Mean ± SEM			
Gender (%)				
Men	19 (15.7)	18 (24.3)	10 (31.2)	0.100^c^
Women	102 (84.3)	56 (75.7)	22 (68.8)	
Age (years)	56.79 ± 0.59	60.95 ± 0.82	60.91 ± 1.51	**<0.001** ^b^ *
Weight (kg)	61.14 ± 0.99	62.83 ± 1.25	58.17 ± 1.50	0.114^b^
Height (cm)	152.60 ± 0.65	153.21 ± 0.93	152.75 ± 1.27	0.977^a^
Body mass index (kg/m^2^) (n = 220)	26.28 ± 0.41	26.72 ± 0.51	24.97 ± 0.64	0.055^a^
Exercise routine (%) (n = 166)				
Exercise	59 (66.3)	31 (56.4)	14 (63.6)	0.486^c^
Do not exercise	30 (33.7)	24 (43.6)	8 (36.4)	
Smoking habit (%) (n = 167)				
Smoking	1 (1.1)	2 (3.6)	1 (4.5)	0.501^c^
Not smoking	88 (98.9)	54 (96.4)	21 (95.5)	
Blood pressure				
Systole (mmHg)	128.91 ± 1.86	128.57 ± 2.55	133.13 ± 3.78	0.397^a^
Diastole (mmHg)	79.78 ± 0.92	77.91 ± 0.99	77.23 ± 1.86	0.215^a^
HbA1c (%)	9.41 ± 0.57	8.24 ± 0.20	7.98 ± 0.28	0.140^a^
Urinary albumin (mcg/dL) (n = 195)	6054.82 ± 1996.65	4339.73 ± 793.70	8483.68 ± 4267.09	0.315^a^
Urinary creatinine (mg/dL) (n = 227)	84.59 ± 5.15	86.05 ± 7.13	90.40 ± 11.86	0.808^a^
Serum creatinine (mg/dL)	0.61 ± 0.01	0.90 ± 0.02	1.70 ± 0.16	**<0.001** ^a^
Albuminuria UACR (mcg/mg Cre) (n = 195)	105.017 ± 28.584	88.675 ± 26.007	119.600 ± 67.434	0.211^a^
Normoalbuminuria (%)	74 (71.2)	42 (62.7)	17 (70.8)	0.488^c^
Albuminuria (%)	30 (28.8)	25 (37.3)	7 (29.2)	

Abbreviations: eGFR, estimated glomerular filtration rate; HbA1c, hemoglobin A1c.

Data were expressed in n (%), Mean ± SEM.

Notes: *p < 0.05 is considered statistically significant; eGFR, estimated glomerular filtration rate; UACR, urine albumin to creatinine ratio; SD, standard deviation; a: Kruskal–Wallis H test; b: one-way ANOVA test; c: chi-square test.

### Trend of oxidative stress markers among groups

Table 2 shows that there were no significant differences (*p*>0.05) in the levels of oxLDL/β_2_GPI complex and 8-iso-PGF2α among the groups. The results showed that oxLDL/β_2_GPI complex was increased linearly from group 1 (0.48 ± 0.03 units/mL) to group 2 (0.54 ± 0.05 units/mL), and then to Group 3 (0.63 ± 0.08 units/mL). In contrary, H_2_O_2_ was shown to be decreased from group 1 (89.99 ± 27.12 μmol/mg creatinine) to group 2 (45.73 ± 11.66 μmol/mg creatinine), and to group 3 (32.83 ± 4.09 μmol/mg creatinine). H_2_O_2_ levels were found to be significantly different among the groups (*p =* 0.003). A unique pattern for 8-iso-PGF2α was found, in which there was an elevation in group 2 (47,356.27 ± 31,982.65 pg/mg creatinine), and then decrease in group 3 (13,172.56 ± 3,335.76 pg/mg creatinine) (Table 2).

**Table 2:** Oxidative stress markers in study subjects.

Characteristic of study subjects	Group 1	Group 2	Group 3	*p*
	eGFR > 89 mL/min/1.73 m^2^ (n = 121)	eGFR 60–89 mL/min/1.73 m^2^ (n = 74)	eGFR < 60 mL/min/1.73 m^2^ (n = 32)	
	Mean ± SE			
H_2_O_2_ (μmol/mg creatinine)	89.99 ± 27.12	45.73 ± 11.66	32.83 ± 4.09	**0.003** ^a^ *
8-iso-PGF2α (pg/mg creatinine)	13,342.32 ± 2,346.87	47,356.27 ± 31,982.64	13,172.56 ± 3,335.76	0.883^a^
OxLDL/β2GPI complex (units/mL)	0.48 ± 0.03	0.54 ± 0.05	0.63 ± 0.08	0.121^a^

Abbreviations: eGFR, estimated glomerular filtration rate; 8-iso-PGF2α, 8-isoprostaglandin F2α; oxLDL/β_2_GPI complex, oxidized low-density lipoprotein/beta-2-glycoprotein-I.

Data were expressed in n (%), mean ± SEM.

Notes: *p* = significance (*p < 0.05 is considered statistically significant); SEM = standard error mean; a = Kruskal–Wallis H tests.

### Correlations between eGFR with oxidative stress markers

**Fig 1:**
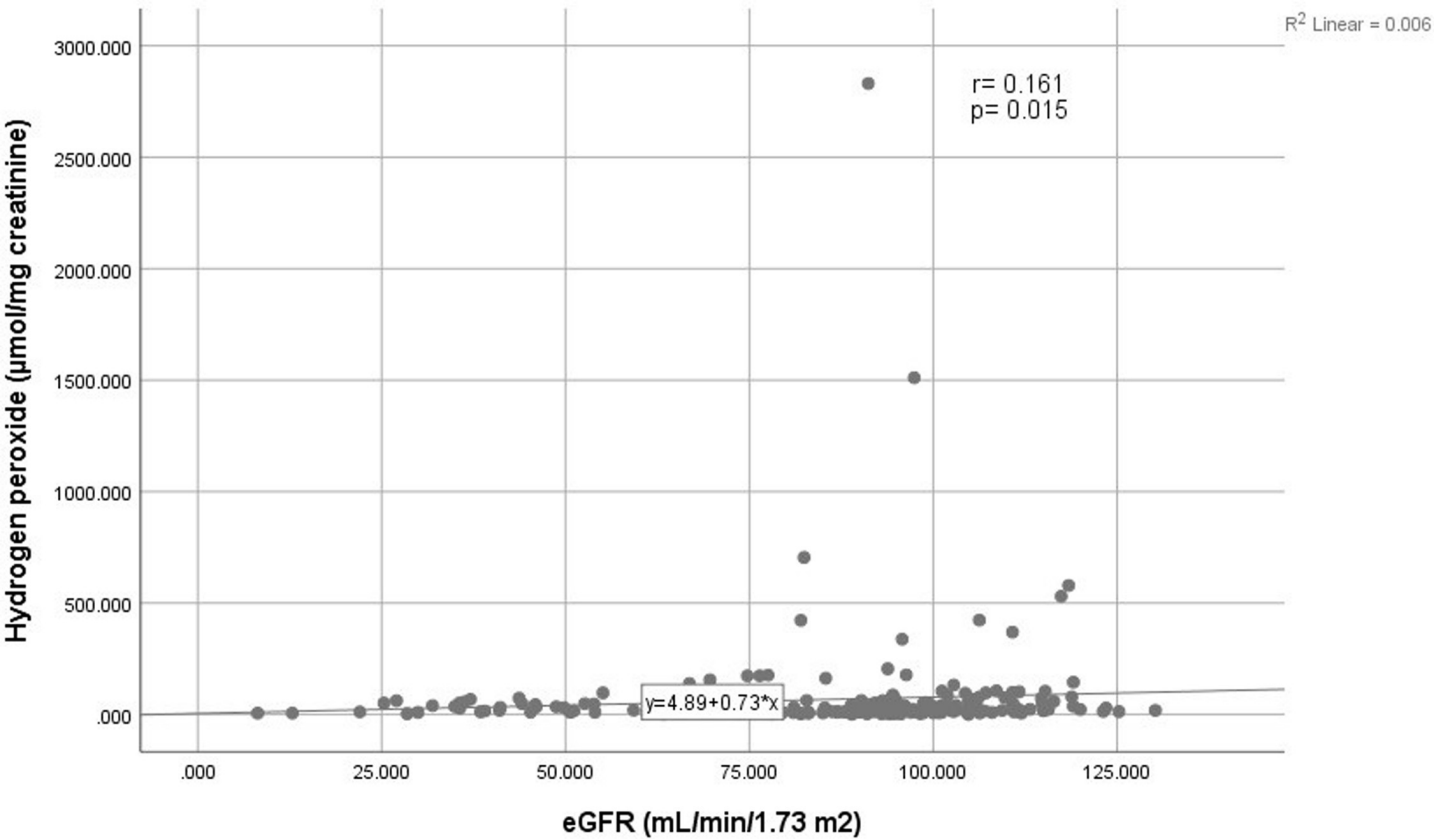
Scatter plot for the correlation between eGFR with urine H_2_O_2_ in total population (n = 227).

Spearman’s Rho correlation test was conducted to determine the correlation between eGFR and H_2_O_2_, 8-iso-PGF2α, and oxLDL/β_2_GPI complex. There was no correlation between markers with eGFR in each group. However, in total population, H_2_O_2_ showed a weak positive correlation with eGFR (*r* = 0.161, *p* = 0.015) (Fig 1). Even after excluding one outlier, the correlation was still significant with the *r* being increased to 0.162. Neither 8-iso-PGF2α levels (*r* = 0.047; *p* = 0.483) nor oxLDL/β_2_GPI complex levels (*r* = -0.093; *p* = 0.164) showed significant correlation with eGFR in total population.

### Correlations between markers

Bivariate correlation between two oxidative stress markers was conducted using Spearman’s Rho Test. Significant correlations was only found in group 2 (eGFR 60–89 ml/min/1.73 m^2^). H_2_O_2_ was correlated with serum oxLDL/β_2_GPI complex (*r* = 0.247; *p* = 0.034) and also with 8-iso-PGF2α (*r* = 0.244; *p* = 0.036). In total population, a significant weak correlation was found between oxLDL/β_2_GPI complex and H_2_O_2_ (*r* = 0.145, *p* = 0.029).

### Multivariate analysis

Multiple linear regression analysis showed that significant factors for increased eGFR were H_2_O_2_ (standardized β = 0.157, p = 0.036), diastolic blood pressure (standardized β = 0.201, p = 0.035), and female (standardized β = 0.191, p = 0.005). Whereas increased systolic blood pressure (standardized β = -0.247, p = 0.011) and age (standardized β = -0.268, p<0.001) were significant factors affecting the decrease of eGFR (Table 3). The model was developed by adjusting UACR, 8-iso-PGF_2_α, OxLDL/β_2_GPI complex, HbA1c, and BMI.

**Table 3:** Factors affecting the eGFR level in T2DM patients (n = 189).

Dependent variable	Adjusted R square	Predictor	Standardized Coefficients β	*p*-value
eGFR (mL/min/1.73 m^2^)	0.182			
		H_2_O_2_ (μmol/mg Cre)	0.157	0.036*
		8-iso-PGF_2_α (pg/mg creatinine)	0.018	0.791
		OxLDL/β_2_GPI complex (units/mL)	-0.109	0.119
		UACR (mcg/mg Cre)	-0.061	0.402
		HbA1c (%)	0.113	0.098
		Systole (mmHg)	-0.247	0.011*
		Diastole (mmHg)	0.201	0.035*
		Age (years)	-0.268	<0.001**
		Gender (female)	0.191	0.005*
		BMI (kg/m^2^)	0.057	0.404

Abbreviations: CI, confidence interval; eGFR, estimated glomerular filtration rate; UACR, urine albumin to creatinine ratio; H_2_O_2_, hydrogen peroxide; 8-iso-PGF_2_α, 8-isoprostaglandin F_2_α; OxLDL/β_2_GPI complex, oxidized low-density lipoprotein/beta-2-glycoprotein-I; HbA1c, hemoglobin A1c; BMI, body mass index;

*statistically significant (p<0.05);

**statistically significant (p<0.001).

## Discussion

There was no significant difference in the proportion of male/female, subjects who routine in exercise, and subjects with smoking habit among three groups. There was also no significant difference in the mean BMI, blood pressure (systole and diastole), HbA1c, urine albumin, urine creatinine, and UACR level. However, there was significant differences in age and of course, serum creatinine. Creatinine clearance and glomerular filtration rate decreases with age [25]. This explains why the prevalence of chronic kidney disease is generally higher in the older age groups.

The eGFR was defined by the volume of filtration entering the Bowman’s capsules per unit of time. The influencing factors of the GFR including capillary blood pressure, interstitial fluid pressure, plasma osmotic pressure, interstitial fluid osmotic pressure, and glomerular surface area. In this study we used the CKD-EPI equation to determine the patient’s eGFR level. Serum creatinine-based equations used to estimate eGFR values include age, sex, race, or weight variables to reflect creatinine formation from muscle mass or diet. There are three known equations, namely Cockroft-Gault (CG), The Modification of Diet in Renal Disease (MDRD), or The Chronic Kidney Disease Epidemiology Collaboration (CKD-EPI). Compared to other creatinine based-eGFR equations, CKD EPI is considered to be the most accurate and precise equation to diagnose CKD, especially for higher GFR values. Therefore, the American Diabetes Association recommends the use of the CKD-EPI equation to estimate the value of the glomerular filtration rate in patients with type 1 or type 2 diabetes mellitus [20, 22]. For UACR measurement, first-morning urine sample was used to avoid biological variations in this study. For diagnosing purpose, this method is more reliable compared to spot urine samples measurement [26].

**Fig 2:**
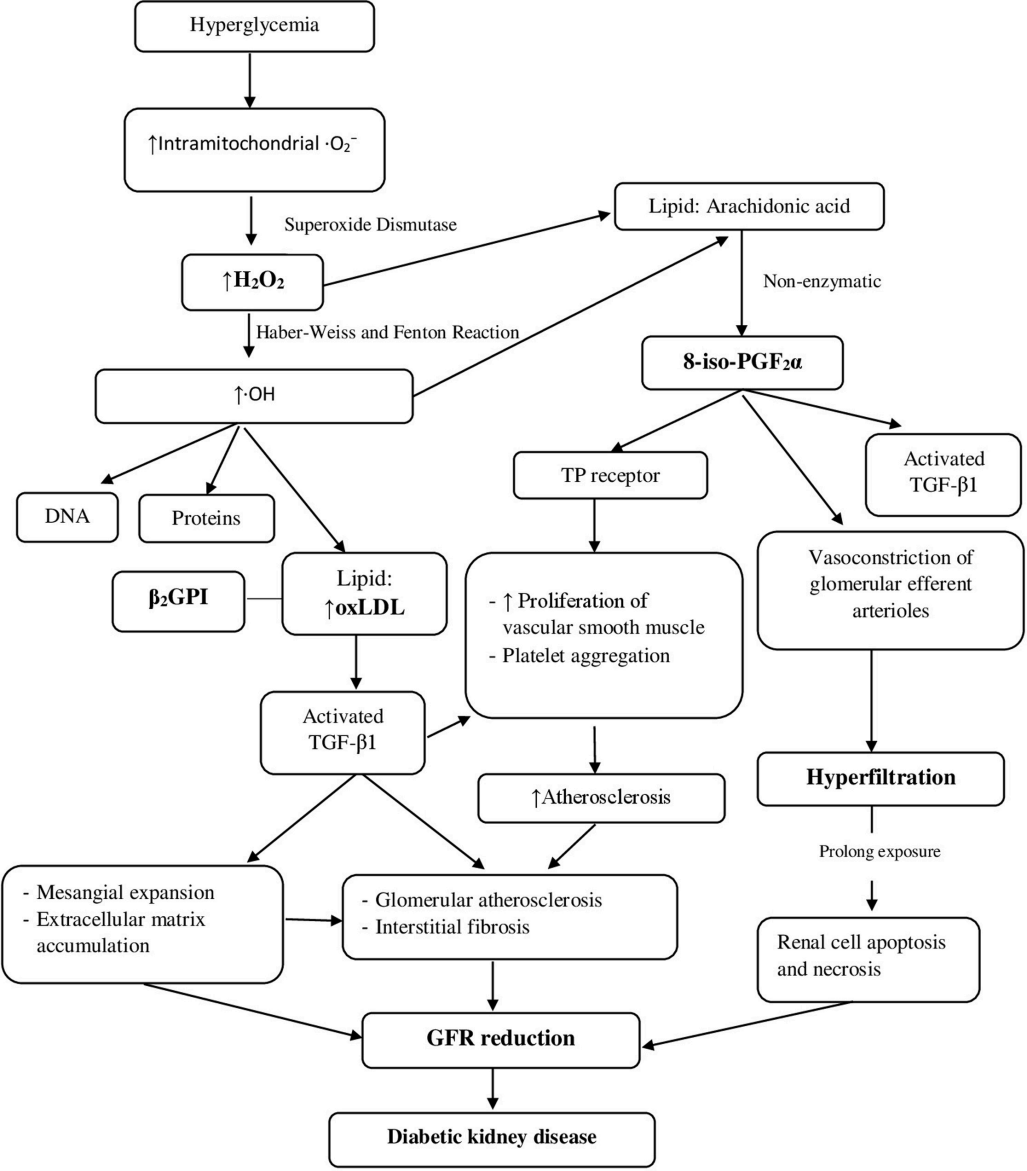
Proposed mechanism of oxidative stress in diabetic kidney disease. O_2_^−^, superoxide anion radical; H_2_O_2_, hydrogen peroxide; OH, hydroxyl radical; DNA, deoxyribonucleic acid; oxLDL, oxidized Low Density Lipoprotein; β_2_GPI, β-2-Glycoprotein-I; TGF-β1, transforming growth factor β1; 8-iso-PGF_2_α, 8-isoprostaglandin F_2_α; TP receptor, thromboxane-prostanoid receptor; GFR reduction, Glomerular Filtration Rate reduction.

Diabetic kidney disease known as diabetic nephropathy is a major chronic microvascular complication in long-standing type 1 and type 2 diabetic patients which can lead to end-stage renal disease (ESRD). The pathophysiological mechanisms in the development of diabetic kidney disease are known to be multifactorial [5, 27, 28]. Hyperglycemia is the initiating event that causes structural and functional changes such as glomerular hyperfiltration, glomerular and tubular epithelial hypertrophy, and microalbuminuria. This process is followed by the development of glomerular basement membrane thickening, accumulation of mesangial matrix, overt proteinuria, and eventually glomerulosclerosis (Fig 2) [27, 28].

Our novel finding in this study was the significant correlation between urinary H_2_O_2_ and eGFR. It means, the concentration of urinary H_2_O_2_ decreased as the CKD stage increased. We also found that urinary H_2_O_2_ had correlation with serum oxLDL/β_2_GPI complex in total population. Moreover, for each group analysis, we only found significant correlation in Group 2 (eGFR 60–89 ml/min/1.73 m^2^). H_2_O_2_ has positive correlation with serum oxLDL/β2GPI complex (r = 0.247; p = 0.034) and also with 8-iso-PGF_2_α (r = 0.244; p = 0.036). However, there could be a bias in Group 2, since this group not only consisted with T2DM patients in G2 stage (also known as silent stage), but also T2DM patients with normal eGFR.

In diabetic kidney disease, a number of sources of ROS are produced through enzymatic and non-enzymatic pathways [29, 30]. H_2_O_2_ is a product of oxidative metabolism as one of the ROS produced by superoxide dismutases (SODs) catalyzation [30]. Several functional enzymes within the mitochondria are particularly susceptible to ROS, leading to altered ATP synthesis, cellular calcium dysregulation, and induction of mitochondrial permeability transition, all of which predispose the cell, including renal cell, to necrosis or apoptosis [29]. It is postulated that localized oxidative stress in renal tissue is a key component in the development of diabetic nephropathy [29]. However, it remains controversy, as to whether this is an early link between hyperglycemia and renal disease or develops because of other primary pathogenic mechanisms [29]. Our study showed that the urinary H_2_O_2_ decreased as CKD stage increased. The production of H_2_O_2_ is a continuation process of intramitochondrial superoxide anion radical (O_2_^−^) production which can further initiate a range of damaging reactions through the production of hydroxyl radical (·OH) through Fenton and Haber-Weiss reaction catalyzed by iron [8]. It is also reported that H_2_O_2_ increases extracellular matrix mRNA through TGF-β in human mesangial cells [31]. Further measurement of serum or tissue H_2_O and ·OH could help to explain this phenomenon.

We did not found a significant positive correlation between eGFR and 8-iso-PGF_2_α, as we reported previously [14]. This could be due to the differences in the nature of the subjects between both studies. Most of the study subjects in previous study was normoalbuminuria G1 and G2 stage, whereas the present study consisted of G1, G2, and G3 stage patients with normoalbuminuria and albuminuria. 8-iso-PGF_2_α is a stable marker of a lipid peroxidation product with a prostaglandin-like structure that is mainly produced in vivo from esterification of arachidonic acid in tissues through non-enzymatic reactions catalyzed by free radicals such as O_2_^−^ and ·OH [32]. A meta-analysis also showed that 8-iso-PGF_2_α could be specifically used as a marker of oxidative stress [31]. Arachidonic acid is one of the phospholipid components that compose cell membranes, including those found in glomerular mesangial cells. Previous cell-culture studies found an increase of isoprostane synthesis in mesangial and endothelial glomerular cells in a high glucose state [33]. 8-iso-PGF_2_α is known to have biological activity as a strong vasoconstrictor [14, 34]. Increasing synthesis of 8-iso-PGF_2_α in the kidneys was reported as increasing the activation of kidney TGF-β in rats with type 1 diabetes [33]. TGF-β is a major pro-fibrotic factor in diabetic nephropathy that could mediate an increase in glomerular permeability to proteins, including albumin [19, 33]. 8-iso-PGF_2_α can interact with the thromboxane prostanoid (TP) receptor of platelets, leading to proteinuria as one of its pathological consequences [35]. In this study, we found that H_2_O_2_ was correlated with 8-iso-PGF2α and also with serum oxLDL/β_2_GPI complex in group 2. An old study but with relevant findings reported that H_2_O_2_ induces 21-aminosteroid-inhibitable F2-isoprostane production in renal epithelial cells that supports the in vivo report that its levels are elevated in ROS-linked renal injury models [36]. Besides direct cell injury, lipid peroxidation by generating F2-isoprostanes, such as 8-iso-PGF2α, may further contribute to renal dysfunction through its vasoconstrictive mechanism [14, 36].

In total population, a significant weak correlation was found between oxLDL/β_2_GPI complex and H_2_O_2_. Research related to the role of oxLDL/β_2_GPI complex in various diseases are still lacking. Previously, the serum oxLDL/β_2_GPI complex were reported to be significantly higher in patients with chronic renal failure, chronic nephritis, and diabetes mellitus than those in healthy individuals [37]. However, in this study, even there was trend of increased oxLDL/β_2_GPI complex as CKD stage increases, the association was not significant. The association of serum oxLDL was formerly reported to have significant contribution to cardiovascular complications in diabetes [38–40]. Recently, Roumeliotis et al. also reported that circulating oxLDL levels amplified the magnitude of the association between proteinuria and progression of diabetic kidney disease [41]. They also found that oxLDL outperformed several confounders and had better accuracy to predict deterioration of eGFR over time than baseline proteinuria [42]. The concepts of oxidative stress and the role of oxLDL in atherosclerosis as a chronic inflammatory process are widely known [38, 40, 43]. However, interestingly, it was reported that oxLDL/β_2_GPI complex, but not free oxidized LDL, was associated with the presence and severity of coronary artery disease [44].

In this study, we found that oxLDL/β_2_GPI complex level was affected by systolic and diastolic blood pressure. Lipid changes and lipoprotein oxidation are known to be related to oxidative stress in diabetes mellitus [39, 40, 45]. The chemical composition of LDL makes these particles susceptible to oxidation by lipid oxidants [16, 17, 43]. Increased level of circulating LDL, especially in oxidized form, is associated with an increased risk of atherosclerosis, including glomerular atherosclerosis [18, 41, 42].

The mechanism of the relationship of hyperglycemia in T2DM to oxidative stress and the forming of atherosclerosis relates to the activity of glucose in monocyte activation. Monocyte activation in high glucose concentrations will increase expression of cytokines, interleukin-1β, and interleukin-6 and increase the release of ·O_2_^−^, which can play a role in glucose-mediated oxidative stress [46, 47]. In addition, hyperglycemia can also cause a decrease in nitric oxide which leads to an increase in oxidative stress [47, 48].

This study has several strengths, one of which is the measurement of several variables simultaneously, such as H_2_O_2_, 8-iso-PGF_2_α, and oxLDL/β_2_GPI complex to seek for oxidative stress biomarkers for early detection of renal damage. We also used a relatively large sample size in various analysis (n = 227). Every analysis was executed as similar with each other as possible by using the same equipment and conditions according to predetermined standard protocols. Due to the unstability of biomarkers in long-term storage, we performed the overall analysis of the biomarkers in less than 1 month after sample collection. Furthermore, samples were stored in temperatures of -20°C and -80°C to maintain their stability. For all biomarkers, we used commercial kit and comply to the instruction guidelines. The data was conducted at minimum duplicate with %CV less than 20. If more than 20, we rerun the analysis. All biomarkers were evaluated for intra assay in previous studies (unpublished) and both quality control low sample (QCL) and low limit of quantification (LLOQ) have shown a %CV less than 15%. In addition, since there was no routine renal function evaluation (eGFR and UACR) for T2DM patients in primary health care, this study is expected to help in examining renal function by measuring eGFR and UACR.

However, there were also limitations in this study. The questionnaire given to subjects was not accurate enough to describe the real conditions. Another limitation of this study was the unavailability of treatment data that might affect markers. However, the study subjects were national health coverage (BPJS) outpatients in primary health care or polyclinic, in which their comorbidities and the treatments were not too varied. Another limitation in this study was the varied age among subjects. To overcome this, we also performed a multivariate analysis. In addition, to minimize bias, we excluded patients who pregnant and breastfeeding women; patients with severe anemia and/or those receiving blood transfusions; patients suffering from heart disease, stroke, impaired liver function, and infectious disease (e.g. tuberculosis); and patients in kidney failure and/or currently undergoing renal replacement therapy.

Despite the role of oxidative stress in early stage of diabetic kidney diseases is still unclear, our findings provide new insight and could lead to further research on the role of H_2_O_2_ in diabetic kidney disease.

## Conclusion

Among three oxidative stress biomarkers studied, only urinary H_2_O_2_ had significant positive correlation with eGFR, even after adjusted by confounding variables. In total population, urinary H_2_O_2_ showed correlation with serum oxLDL/β_2_GPI complex. This finding could lead to further research on urinary H_2_O_2_ for early detection and research on novel therapies of diabetic kidney disease.

## Supporting information

S1 File Raw data. (PDF)

## Abbreviations

BMI: body mass index
CG: cockroft-gault
CI: confidence interval
CKD-EPI: chronic kidney disease epidemiology collaboration
DA: delta absorbance
DNA: deoxyribonucleic acid
eGFR: estimated glomerular filtration rate
ELISA: enzyme-linked immunosorbent assay
ESRD: end-stage renal disease
FOX-1: ferrous ion oxidation xylenol orange method 1
HbA1c: hemoglobin A1c
HRP-SA: horseradish peroxidase-conjugated streptavidin
H_2_O_2_: Hydrogen peroxide
IDF: International Diabetes Federation
LDL: low-density lipoprotein
MDRD: modification of diet in renal disease
OR: odds ratio
OxLDL/β_2_GPI complex: oxidized low-density lipoprotein/beta-2-glycoprotein-I
ROS: reactive oxygen species
SOD: superoxide dismutases
TGF-β: transforming growth factor beta
TMB: tetramethylbenzidine
T2DM: type 2 diabetes mellitus
8-iso-PGF_2_α: 8-isoprostaglandin F_2_α
