# Supplementary material for: Role of urinary H2O2, 8-iso-PGF2α, and serum oxLDL/β2GP1 complex in the diabetic kidney disease
Source: PLoS One. 2022 Apr 5;17(4):e0263113. doi: 10.1371/journal.pone.0263113 (PMC8982868; doi:10.1371/journal.pone.0263113)
Supplement: S1 File — (PDF) [file pone.0263113.s001.pdf]

eGFR	UACR (mcg/mg)	isoprostan	11dTXB2 (pg/mgkreatinin)	oxLDL complexes	hydrogen peroxyde
104.726	3.7	3548.567	1175.179	0.121	50.532
103.106	0.0	5596.152	47.017	0.229	41.263
99.553	10.5	7036.070	245.696	0.095	45.636
78.805	12.4	5155.981	1493.479	0.322	11.082
89.000	17.1	3588.776	969.641	0.343	12.218
100.780	0.0	4356.223	1174.531	0.840	7.444
110.747	0.0	7748.765	98.967	0.371	29.193
83.068	11.9	1854.110	1595.958	2.282	7.923
65.549	3.6	2636.901	1461.851	0.208	9.997
113.162	0.0	6199.357	90.192	0.216	23.653
28.457	556.2	6423.148	79.665	0.534	3.584
130.202	0.0	11309.682	425.152	0.165	18.800
94.313	7.3	2717.068	1583.422	0.430	5.743
101.177	29.1	4725.598	316.720	0.360	10.371
78.411	0.0	7139.849	17.043	0.150	17.697
94.713	10.3	2527.335	701.287	0.307	5.506
90.079	2.3	4397.328	100.865	0.614	25.590
102.821	0.0	3395.240	175.831	0.136	14.350
75.229	26.3	2826.977	944.173	0.375	3.782
74.205	3.8	3223.316	685.888	0.348	23.172
68.064	115.2	39541.554	1319.392	0.293	61.512
109.363	21.6	9192.978	226.050	0.635	18.986
83.009	39.6	4171.667	172.376	1.324	9.720
101.177	10.3	8389.947	216.988	0.872	35.168
96.052	7.8	4948.656	457.571	1.032	41.108
77.706	60.7	4108.680	634.551	1.224	8.741
75.255	14.8	4741.946	1758.813	0.293	5.082
22.037	466.4	2711.208	389.881	1.534	11.690
59.263		5447.733	71.608	1.174	19.665
92.505		9636.697	357.304	0.558	36.346
114.806		2649.576	1572.806	0.522	42.775
104.399		3154.757	407.436	1.037	14.941
68.314		2250.081	953.435	0.260	19.238
111.635		7956.459	474.102	0.512	13.163
29.932		1932.669	399.684	0.921	9.677
50.588		8432.538	221.374	0.708	10.744
51.128		39050.971	163.404	0.278	16.706
98.630		1006.100	448.031	0.333	7.391
8.127		2385.307	39.613	1.891	5.954
106.313		2664.683	176.734	0.250	6.570
73.667		6681.242	684.649	0.335	3.295
104.740		1251.624	735.959	0.248	4.497
104.723		3522.444	624.386	0.221	7.533
65.741		2686.483	470.973	0.346	7.230
63.321		2170.868	467.929	0.499	1.427

98.237		3031.455	762.125	0.500	5.240
98.160		5036.624	891.539	0.185	6.645
105.877		52081.611	46.112	0.500	42.616
115.227		3568.163	269.459	0.200	104.022
45.241		3006.304	87.082	0.315	10.315
63.576		6820.084	17.877	0.549	22.959
68.853		5285.171	37.213	0.307	21.064
119.988		4351.530	259.118	0.368	22.383
95.038		6227.221	222.260	0.637	18.227
98.974		4940.697	66.302	0.192	53.827
106.986		6115.919	465.985	0.771	15.812
98.630		5675.345	126.483	0.185	49.302
76.586		3532.656	246.100	0.215	42.211
50.756		3806.590	1002.443	0.156	12.381
31.913		7913.564	141.368	0.329	39.997
73.018	0.0	1800.091	1729.278	0.460	7.377
115.615	2.7	5524.254	5273.787	0.505	36.940
81.051	0.0	6480.315	9529.703	1.553	17.770
67.738	0.0	1038.404	3888.063	1.016	16.475
53.875	50.6	4517.928	4615.477	0.751	47.222
76.399	0.0	3649.763	5436.048	0.649	174.132
95.176	40.7	4296.164	3028.653	0.448	49.474
92.505	93.8	6251.390	5803.178	0.626	49.240
102.789	72.0	1544.012	2369.786	1.052	132.393
88.921	17.0	2106.134	342.053	0.420	28.592
34.946	0.0	5873.318	614.895	0.513	36.000
36.257	0.0	6583.316	2627.388	0.740	56.962
90.218	67.0	3391.674	3477.093	0.355	62.777
82.004	126.8	8015.915	5320.706	1.023	422.727
108.597	528.3	4600.798	9017.982	0.759	106.341
102.070	78.1	1873.406	4575.913	0.511	34.483
69.635	0.0	2528.943	2498.978	0.465	155.000
104.973	1.1	1312.637	4448.469	0.705	60.000
75.281	2.3	1909.713	7338.177	0.507	96.825
67.401	0.0	3543.503	4815.116	1.997	26.582
111.635	6.9	666.344	4879.692	0.336	20.471
93.900	0.0	60.476	5053.347	0.963	26.471
106.229	0.0	2605.040	1658.347	0.571	78.355
77.925	17.8	1996.590	1953.487	1.126	21.512
88.824	0.0	1625.201	2655.032	0.478	41.466
119.059	2.9	3346.440	5969.294	0.582	145.122
25.322	15.3	3773.329	895.237	1.376	50.829
123.506	82.2	2703.134	4542.672	0.604	29.240
27.018	0.0	1442.873	1985.212	0.408	62.130
100.253	7.4	2462.643	4916.193	0.787	32.574
95.439	27.9	2274.521	12267.904	0.585	22.411
86.232	33.9	1771.684	2353.112	0.600	13.176
100.850	2.3	3026.444	16145.925	0.571	21.698

102.556	13.4	2352.112	9090.477	0.921	16.338
53.998	0.0	1148.824	23925.761	0.551	10.590
88.910	10.7	1060.670	5457.098	0.432	1.212
110.980	0.0	21496.723	2588.457	0.349	12.548
41.022	134.2	33953.904	3123.561	0.425	18.791
74.728	0.0	716217.571	27263.351	0.408	173.875
110.853	50.1	48079.298	11591.101	0.341	48.922
118.408	79.7	5439.323	196.452	0.849	578.806
43.689	0.0	14079.401	4804.753	0.475	73.285
118.835	0.0	42733.202	11019.741	0.477	79.147
102.512	19.3	22191.355	4685.029	0.559	27.833
81.051	14.8	17968.290	4623.650	0.719	31.884
35.565	0.0	34063.850	6921.649	0.434	50.933
100.832	8.5	27049.513	6576.005	0.743	25.698
115.677	15.2	40383.197	10565.004	0.540	23.562
48.724	3.7	15930.784	4623.312	0.576	35.253
73.195	91.7	4179.051	25347.032	1.228	17.075
72.394	0.0	15952.556	43134.071	0.859	60.426
97.203	29.3	1749.094	29631.953	0.634	24.145
93.157	0.0	2995.926	57197.774	1.327	62.432
104.351	0.0	3833.618	294729.036	1.163	96.026
101.350	0.0	849.174	42550.322	0.365	26.034
116.396	219.7	1742.806	6856.624	1.876	59.155
92.089	0.0	4541.310	154182.004	0.352	51.206
85.251	0.0	1906.830	201581.374	0.449	29.928
35.631	13.5	1913.354	388.877	0.404	28.593
91.173	1358.1	6599.302	139817.930	2.274	2830.769
44.097	0.0	1473.305	39094.130	0.244	48.638
109.719	30.3	1231.423	1393.560	0.316	75.610
101.177	0.0	2273.745	2511.158	0.308	105.420
46.014	22.6	1709.612	479.051	0.898	33.898
94.847	48.7	34891.719	0.000	0.330	68.649
94.050	0.0	3237.266	4181.888	0.479	63.872
114.706	0.0	4094.025	6986.995	0.303	72.519
117.398	0.0	7038.605	46425.054	0.545	530.556
38.438	0.0	1236.649	732.124	0.408	12.476
93.814	1549.2	9987.068	10876.532	0.395	205.283
95.768	0.0	3518.803	42297.180	0.480	337.956
82.437	1481.6	7420.394	25583.906	0.372	704.444
96.337	306.1	3481.055	1681.008	0.430	177.922
45.895	0.0	10638.945	3938.392	0.426	44.891
111.692	27.7	3771.742	8443.271	0.501	102.194
94.509	0.0	3466.971	13100.977	0.450	87.811
110.774	0.0	4328.187	40502.660	0.347	369.828
110.774	0.0	2730.570	490.114	0.293	99.784
66.843	0.0	2600.820	9557.718	0.432	138.732
97.412	0.0	4862.973	49027.777	0.308	1510.714
55.085	0.0	5634.321	377.908	1.600	97.354

95.071	0.0	3767.724	4036.822	0.550	9.226
82.799	45.2	6369.670	10405.274	0.360	63.026
37.072	51.4	598.319	9709.404	0.350	68.059
93.157	1539.9	3966.840	642.258	0.360	50.799
101.179	0.0	3483.749	5407.097	0.330	41.918
90.932	0.0	32540.195	15943.917	0.500	40.000
119.059	0.0	14923.682	2655.914	0.280	37.229
114.998	31.9	41203.001	615.192	0.660	18.632
39.025	0.0	52326.682	1972.269	0.310	15.926
104.792	425.9	31949.193	14687.613	0.670	0.968
88.782	471.5	2273791.593	57926.498	0.230	25.455
123.106	0.0	12876.268	92.889	0.620	14.091
95.593	16.4	91717.972	11466.335	0.400	2.308
106.269	1266.7	121254.250	38203.332	0.390	423.387
101.927	0.0	34750.525	19768.062	0.300	85.556
98.409	0.0	190942.779	142255.075	0.480	23.016
94.775	0.0	41667.580	6592.963	0.410	5.556
63.947	0.0	18963.560	8774.374	0.400	33.603
107.150	612.5	121121.846	19743.982	0.180	97.354
67.170	0.0	51350.051	24080.962	0.320	103.846
52.607	1516.9	31909.233	11574.728	0.340	48.795
93.667	277.4	46952.446	8868.693	0.310	38.038
64.050	114.5	15911.126	6215.231	1.000	38.910
41.113	1.8	87207.366	7240.354	0.420	31.928
105.087	0.0	49404.388	8213.541	0.200	70.741
49.786	0.8	20109.757	2305.937	0.340	30.982
125.253	0.0	1183.436	2504.044	0.390	13.540
79.522	22.0	9217.307	7896.852	0.320	9.605
12.848	37.0	4286.775	7930.699	0.440	6.257
111.765	0.0	8508.848		0.300	18.321
85.384	422.2	25135.582	49607.673	0.410	162.963
77.535	102.5	16091.604	60398.803	0.350	176.744
103.000	104.4	5631.550		0.970	19.618
90.000	11.8	1981.280		0.110	11.396
106.000	17.0	6695.770		0.150	30.724
105.000	36.1	13139.750		0.150	18.249
103.000	28.3	1357.840		0.150	23.676
111.000	10.8	13006.800		0.180	18.442
108.000	98.2	4428.960		0.240	9.606
92.000	481.6	6550.200		0.180	27.173
91.000	27.4	2514.570		0.140	3.432
112.000	10.2	3451.190		0.230	12.933
96.000	6.6	3000.470		0.150	24.115
111.000	45.6	3129.260		0.070	34.737
92.000	6.0	4287.780		0.330	12.980
115.000	12.3	7501.950		0.370	16.778
94.000	10.8	3372.500		0.270	2.627
103.000	481.6	16274.190		0.260	20.921

98.000	9.8	9244.650		0.440	10.314
91.000	9.1	7761.600		0.770	9.282
98.000	13.9	2659.360		0.110	11.483
95.000	16.2	8762.420		1.000	16.522
93.000	15.4	10636.240		0.800	2.535
100.000	0.0	33828.890		0.180	7.078
101.000	9.9	5063.610		0.670	12.232
95.000	257.8	2351.970		0.270	22.395
112.000	115.6	6604.510		0.190	6.658
97.000	43.2	3119.420		0.130	8.966
87.000	630.9	5003.940		1.990	14.148
81.000	26.2	7669.390		0.210	7.850
69.000	465.1	2798.490		0.380	10.262
89.000	51.2	9293.440		0.940	4.254
85.000	19.6	5289.440		0.320	10.124
78.000	125.4	1978.040		0.200	2.919
82.000	106.6	2041.960		0.390	4.988
73.000	23.3	20185.360		0.790	25.354
82.000	37.0	779.900		0.070	3.000
66.000	115.1	8216.350		0.230	6.742
67.000	8.8	6546.620		0.150	10.388
66.000	8.0	4505.400		0.120	7.127
85.000	12.6	10184.490		0.770	7.383
77.000	15.8	3050.170		0.150	14.893
66.000	25.4	2163.150		0.180	10.525
89.000	299.9	6373.880		0.820	12.995
71.000	112.5	9172.580		0.140	9.884
83.000	7.2	9122.150		0.190	6.575
66.000	9.0	5447.750		0.500	8.326
87.000	9.7	9664.960		0.200	10.232
88.000	88.9	7620.580		0.450	12.930
89.000	97.4	10692.580		0.130	5.305
69.000	101.0	6261.510		0.140	7.229
61.000	238.9	3889.290		0.260	6.504
77.000	4.9	2602.860		0.630	5.111
65.000	16.4	4265.120		0.170	52.875
88.000	0.0	8649.750		0.110	10.114
69.000	4.5	1875.770		1.010	14.491

Data	Jenis Kelamin (1=laki2,2=perempuan)	Usia (tahun)	BB (kg)	TB (cm)	TB (m)	BMI	BSA
2015 sitanala	2	65	50.0	160.0	1.600	19.531	1.501
	2	54	59.0	152.0	1.520	25.537	1.552
	1	64	39.0	154.0	1.540	16.445	1.314
	2	65	76.0				
	2	65		155.0	1.550		
	2	55	58.5	151.0	1.510	25.657	1.539
	2	47	57.0	158.0	1.580	22.833	1.573
	2	49	47.0	160.0	1.600	18.359	1.462
	2	65	58.0	158.0	1.580	23.233	1.584
	2	55	61.0	156.0	1.560	25.066	1.604
	2	51	44.0	148.0	1.480	20.088	1.344
	2	51	68.0	147.5	1.475	31.255	1.613
	2	63	72.0	152.0	1.520	31.163	1.689
	2	53	82.0	153.0	1.530	35.029	1.793
	1	60	68.0	169.0	1.690	23.809	1.780
	2	61	71.0				
	1	63	79.0	170.0	1.700	27.336	1.905
	2	50	52.0				
	2	57	51.0	148.0	1.480	23.283	1.431
	2	63	67.0	146.0	1.460	31.432	1.591
	1	63	68.0	164.0	1.640	25.283	1.742
	2	54	73.0	158.0	1.580	29.242	1.747
	2	41	59.0	156.0	1.560	24.244	1.581
	2	53	94.0	150.0	1.500	41.778	1.873
	1	52	77.0	161.0	1.610	29.706	1.812
	2	67	68.0	146.0	1.460	31.901	1.601
	2	61	57.0	148.0	1.480	26.023	1.500
	1	60	65.0	164.0	1.640	24.167	1.709
	2	65	62.0	141.0	1.410	31.186	1.501
	2	63	62.0	147.5	1.475	28.498	1.551
	2	54	44.5	147.5	1.475	20.454	1.347
	2	53	64.0	155.0	1.550	26.639	1.629
	1	51	56.0	170.0	1.700	19.377	1.646
	2	52	85.0	160.0	1.600	33.203	1.881
	2	63	47.0	149.0	1.490	21.170	1.389
	1	61	71.0	160.0	1.600	27.734	1.742
	2	59	58.0	148.0	1.480	26.479	1.511
	1	63	64.0	169.0	1.690	22.408	1.735
	2	56	76.0	148.0	1.480	34.697	1.695
	1	65	64.0	162.0	1.620	24.387	1.682
	2	62	78.0	148.5	1.485	35.371	1.718
	2	51	49.0	144.0	1.440	23.630	1.379
	1	48	47.0	167.0	1.670	16.853	1.508
	1	65	43.0	151.5	1.515	18.735	1.353
	1	55	75.0	165.0	1.650	27.548	1.824

	2	52	53.0	149.5	1.495	23.713	1.465
	1	60	70.0	166.0	1.660	25.403	1.779
	2	51	60.5	152.5	1.525	26.015	1.572
	1	49	63.0	160.0	1.600	24.609	1.656
	2	48	46.5	144.0	1.440	22.425	1.348
	2	55	61.0	160.0	1.600	23.828	1.633
	2	58	64.0				
	1	62	61.0	164.0	1.640	22.680	1.663
	2	45	53.0				
	2	63	59.0				
	1	57	77.0	165.0	1.650	28.283	1.844
	1	63	59.5	160.0	1.600	23.242	1.616
	1	65	71.0	174.0	1.740	23.451	1.852
	1	56	57.0	162.0	1.620	21.719	1.601
	2	51	53.0	148.0	1.480	24.196	1.454
**Data 2016**	1	50	74.0	164.0	1.640	27.513	1.805
2016 albumin urinnya dalam mg/dl	2	53	54.0	148.0	1.480	24.653	1.466
	2	61	62.0	147.0	1.470	28.692	1.547
	2	53	69.0	155.0	1.550	28.720	1.682
	1	75	59.0	163.0	1.630	22.206	1.632
	2	63	49.0	150.0	1.500	21.778	1.420
	2	61	86.0	156.0	1.560	35.339	1.856
	2	63	57.5	146.0	1.460	26.975	1.491
	2	56	61.0	145.0	1.450	29.013	1.521
	2	50	67.5	149.0	1.490	30.404	1.619
	2	67	47.0	151.0	1.510	20.613	1.402
	2	71	50.0	148.0	1.480	22.827	1.419
	2	57	64.0	154.0	1.540	26.986	1.622
	2	66	43.0	140.0	1.400	21.939	1.278
	2	55	58.0	148.0	1.480	26.479	1.511
	2	57	63.0	160.0	1.600	24.609	1.656
	2	68	52.0	146.0	1.460	24.395	1.428
	2	45	67.0	148.0	1.480	30.588	1.607
	2	51	62.0	156.0	1.560	25.477	1.615
	1	57	61.0	154.0	1.540	25.721	1.589
	2	52	60.0	156.0	1.560	24.655	1.593
	1	67	75.0	160.0	1.600	29.297	1.783
	2	64	50.5	148.0	1.480	23.055	1.425
	2	54	46.5	146.0	1.460	21.815	1.362
	1	62	70.0	155.0	1.550	29.136	1.692
	2	51	74.0	144.0	1.440	35.687	1.643
	2	60	62.0	160.0	1.600	24.219	1.645
	2	53	75.5	147.0	1.470	34.939	1.682
	2	61	55.0	155.0	1.550	22.893	1.528
	2	62	48.0	142.0	1.420	23.805	1.353
	2	65	45.0	144.0	1.440	21.701	1.330
	1	70	88.0	162.0	1.620	33.531	1.926
	2	62	64.0	155.0	1.550	26.639	1.629

	2	54	50.0	144.0	1.440	24.113	1.391
	2	65	71.0	151.0	1.510	31.139	1.671
	2	70	64.0	145.0	1.450	30.440	1.552
	2	51	62.0	156.0	1.560	25.477	1.615
	2	67	53.5	150.0	1.500	23.778	1.474
	2	62	66.0	151.0	1.510	28.946	1.620
	2	53	70.0	165.0	1.650	25.712	1.771
	2	59	43.0	147.0	1.470	19.899	1.324
	2	70	51.0	155.0	1.550	21.228	1.479
	2	46	50.0	152.0	1.520	21.641	1.446
	2	58	67.0	149.0	1.490	30.179	1.614
	2	61	77.0	160.0	1.600	30.078	1.803
	1	73	71.0	159.0	1.590	28.084	1.734
	1	65	64.0	160.0	1.600	25.000	1.667
	2	54	49.0	146.0	1.460	22.987	1.393
	1	68	63.0	160.0	1.600	24.609	1.656
	2	55	52.0	150.0	1.500	23.111	1.457
	2	49	67.0	151.0	1.510	29.385	1.630
	2	58	70.0	161.0	1.610	27.005	1.740
	2	62	65.5	149.0	1.490	29.503	1.599
	2	58	54.0	140.0	1.400	27.551	1.408
	2	50	56.5	152.0	1.520	24.455	1.523
	2	47	58.5	151.0	1.510	25.657	1.539
	2	65	61.5	147.0	1.470	28.460	1.541
	2	56	64.0	153.0	1.530	27.340	1.614
	2	62	51.0	149.0	1.490	22.972	1.438
	2	70	48.5	150.0	1.500	21.556	1.414
	2	58	62.0	151.0	1.510	27.192	1.577
	2	50	57.0	150.0	1.500	25.333	1.515
	2	53	65.0	152.0	1.520	28.134	1.617
	1	64	65.0	165.0	1.650	23.875	1.716
	2	57	48.5	144.0	1.440	23.389	1.373
	2	62	58.0	150.0	1.500	25.778	1.526
	2	42	65.0	162.0	1.620	24.768	1.693
	2	53	79.0	154.0	1.540	33.311	1.773
	1	49	54.0	158.0	1.580	21.631	1.537
	2	61	59.0	152.0	1.520	25.537	1.552
	2	63	59.0	154.0	1.540	24.878	1.567
	2	63	79.0	150.0	1.500	35.111	1.740
	2	50	50.0	153.0	1.530	21.359	1.453
	1	33	74.0	160.0	1.600	28.906	1.773
	2	51	62.0	150.0	1.500	27.556	1.570
	2	62	65.0	161.0	1.610	25.076	1.686
	2	56	61.0	145.0	1.450	29.013	1.521
	2	56	62.0	154.0	1.540	26.143	1.600
	2	66	70.0	150.0	1.500	31.111	1.653
	1	57	58.0	156.0	1.560	23.833	1.570
	2	72	46.0	142.0	1.420	22.813	1.329

	1	66	68.0	160.0	1.600	26.562	1.711
	1	72	70.0	165.0	1.650	25.712	1.771
	2	62	55.0	157.0	1.570	22.313	1.542
	2	62	66.0	153.0	1.530	28.194	1.635
	2	66	41.0	148.0	1.480	18.718	1.304
	2	63	47.0	149.0	1.490	21.170	1.389
	2	51	58.0	140.0	1.400	29.592	1.451
	1	60	63.0	157.0	1.570	25.559	1.633
	2	57	60.0	143.0	1.430	29.341	1.495
	2	58	36.0	140.0	1.400	18.367	1.185
	2	64	56.0	150.0	1.500	24.889	1.503
	2	62	58.0	156.0	1.560	23.833	1.570
	2	59	64.0	145.0	1.450	30.440	1.552
	2	65	61.0	155.0	1.550	25.390	1.596
	2	58	56.0	150.0	1.500	24.889	1.503
	2	63	50.0	141.0	1.410	25.150	1.370
	2	70	53.0	153.0	1.530	22.641	1.490
	2	63	64.0	145.0	1.450	30.440	1.552
	2	56	56.0	150.0	1.500	24.889	1.503
	2	56	44.0	143.0	1.430	21.517	1.311
	2	64	56.0	161.0	1.610	21.604	1.582
	2	54	52.0	150.0	1.500	23.111	1.457
	2	74	57.5	146.0	1.460	26.975	1.491
	1	67	56.0	142.0	1.420	27.772	1.445
	2	57	64.0	151.0	1.510	28.069	1.599
	2	57	59.0	151.0	1.510	25.876	1.544
	2	51	57.0	158.0	1.580	22.833	1.573
	1	55	66.0	159.0	1.590	26.107	1.681
	2	57	61.5	145.0	1.450	29.251	1.526
	2	60	59.0	154.0	1.540	24.878	1.567
	2	58	76.0	146.0	1.460	35.654	1.678
	1	67	46.0	168.0	1.680	16.298	1.501
**Data 2019**	2	54	67.0	148.0	1.480	30.588	
	1	45	56.0	168.0	1.680	19.841	
	2	48	60.0	144.0	1.440	28.935	
	2	50	57.0	140.0	1.400	29.082	
	2	56	66.0	152.0	1.520	28.566	
	2	51	70.0	151.0	1.510	30.700	
	2	56	55.0	150.0	1.500	24.444	
	2	45	69.0	158.0	1.580	27.640	
	2	70	53.0	148.0	1.480	24.196	
	2	53	52.0	157.0	1.570	21.096	
	2	42	71.0	157.0	1.570	28.804	
	2	48	82.0	164.0	1.640	30.488	
	2	65	67.0	146.0	1.460	31.432	
	2	46	94.0	150.0	1.500	41.778	
	1	68	60.0	160.0	1.600	23.437	
	2	53	78.0	146.0	1.460	36.592	

	2	68	58.0	145.0	1.450	27.586	
	2	61	66.0	157.0	1.570	26.776	
	2	65	45.0	144.0	1.440	21.701	
	2	59	74.0	149.0	1.490	33.332	
	2	61	55.0	150.0	1.500	24.444	
	1	55	76.0	170.0	1.700	26.298	
	2	52	54.0	153.0	1.530	23.068	
	2	69	40.0	139.0	1.390	20.703	
	2	56	71.0	154.0	1.540	29.938	
	2	57	44.0	146.0	1.460	20.642	
	2	60	81.0	155.0	1.550	33.715	
	1	68	52.0	168.0	1.680	18.424	
	1	59	83.0	162.0	1.620	31.626	
	2	53	71.0	156.0	1.560	29.175	
	2	74	52.0	153.0	1.530	22.214	
	2	71	67.0	153.0	1.530	28.621	
	2	64	45.0	147.0	1.470	20.825	
	2	68	60.0	153.0	1.530	25.631	
	2	64	56.0	147.0	1.470	25.915	
	2	60	61.0	151.0	1.510	26.753	
	2	68	57.0	154.0	1.540	24.034	
	2	72	49.0	143.0	1.430	23.962	
	2	55	63.0	157.0	1.570	25.559	
	2	70	60.0	153.0	1.530	25.631	
	2	60	50.0	144.0	1.440	24.113	
	1	48	79.0	165.0	1.650	29.017	
	2	66	65.0	148.0	1.480	29.675	
	2	73	63.0	148.0	1.480	28.762	
	2	65	68.0	140.0	1.400	34.694	
	2	57	70.0	152.0	1.520	30.298	
	2	69	60.0	144.0	1.440	28.935	
	2	59	61.0	152.0	1.520	26.402	
	2	60	79.0	154.0	1.540	33.311	
	2	53	57.0	147.0	1.470	26.378	
	1	54	62.0	156.0	1.560	25.477	
	2	58	53.0	143.0	1.430	25.918	
	2	53	51.0	144.0	1.440	24.595	
	1	66	82.0	167.0	1.670	29.402	

Lama Durasi DM	Serum (mg/dL)R/M	Sistole	Diastole	HbA1c	KreatininSerum (mg/dL)	Urin albumin (mg/L)	Urin kreatinin (g/l)	Urin albumin (mcg/dl)
	0.46	165	89	4.7		1.9	0.514	188
	0.61	127	96	6.8		0.0	0.265	0
	0.74	97	65	5.3		1.9	0.179	188
	0.79	152	76	7.1		8.9	0.719	895
	0.71	183	93	5.8		13.7	0.799	1365
	0.64	151	93	6.5		0.0	0.977	0
	0.57	160	122	6.5		0.0	0.268	0
	0.83	170	91	7.1		16.0	1.345	1601
	0.92	117	87	7.4		6.6	1.845	659
	0.45	166	81	9.6		0.0	0.491	0
	1.99	114	69	8.6		251.4	0.452	25141
	0.32	147	94	12.4		0.0	0.174	0
	0.66			6.0		8.9	1.219	895
	0.66	154	76	7.5		27.8	0.954	2778
	1.05	150	95	8.3		0.0	0.255	0
	0.68	142	85	9.8		8.9	0.871	895
	0.92	172	105	75.0		1.9	0.834	188
	0.67	111	60	6.7		0.0	0.661	0
	0.86	185	95	7.9		13.7	0.520	1365
	0.84	121	66	5.9		1.9	0.502	188
	1.16	110	80	6.5		32.5	0.282	3249
	0.51	151	90	10.3		18.4	0.852	1836
	0.87	142	94	9.1		49.0	1.238	4896
	0.66	142	102	11.1		4.2	0.411	424
	0.93			11.0		6.6	0.848	659
	0.79	163	76	7.9		41.9	0.690	4190
	0.84	152	72	8.0		16.0	1.079	1601
	3.00	195	112	6.4		592.8	1.271	59275
	1.00	162	81	11.8			0.619	
	0.70	196	80	14.0			0.415	
	0.44	116	60	8.0	0.44		0.626	
	0.60	116	88	6.4	0.60		0.637	
	1.24	146	86	6.6	1.24		1.373	
	0.50	156	85	8.1	0.50		0.430	
	1.78	113	72	8.2	1.78		0.923	
	1.50	157	84	7.4	1.50		0.231	
	1.17			7.0	1.17		0.029	
	0.77	150	80	8.6	0.77		0.891	
	5.45			9.3	5.45		0.185	
	0.62			8.7	0.62		1.160	
	0.85	132	78	7.1	0.85		0.995	
	0.62	160	75	6.4	0.62		1.035	
	0.86	116	67	6.5	0.86		1.268	
	1.18	134	60	6.2	1.18		0.315	
	1.29	119	78	8.2	1.29		1.233	

	0.71	140	80	7.5	0.71		1.000	
	0.82	148	77	5.6	0.82		0.477	
	0.60	165	119	6.3	0.60		0.030	
	0.67	130	81	7.1	0.67		0.307	
	1.38	109	64	7.8	1.38		0.461	
	1.00	164	100	14.0	1.00		0.206	
	0.92	159	71	10.4	0.92		0.214	
	0.73	119	78	6.0	0.73		0.317	
	0.76	169	92	5.7	0.76		0.921	
	0.57	199	88	5.6	0.57		0.272	
	0.70	116	73	8.0	0.70		0.720	
	0.77	190	73	7.2	0.77		0.373	
	1.04	165	77	6.1	1.04		0.451	
	1.54	123	64	5.1	1.54		0.836	
	1.81	131	81	6.7	1.81		0.153	
	1.16	100	70	5.7	1.16	0.0	1.281	0
	0.44	130	80	8.4	0.44	1.5	0.563	152
	0.79	180	100	8.0	0.79	0.0	0.603	0
	0.96	120	80	8.8	0.96	0.0	2.193	0
	1.29	140	70	9.9	1.29	34.5	0.681	3448
	0.82	110	80	7.2	0.82	0.0	0.665	0
	0.67	140	90	8.0	0.67	24.3	0.598	2434
	0.70	140	80	6.4	0.70	64.9	0.692	6491
	0.59	130	80	10.9	0.59	123.2	1.712	12323
	0.78	120	80	10.8	0.78	24.3	1.431	2434
	1.53	120	70	7.7	1.53	0.0	0.105	0
	1.45	140	70	6.1	1.45	0.0	0.166	0
	0.74	110	80	11.0	0.74	70.0	1.044	6998
	0.76	110	70	8.5	0.76	11.7	0.092	1166
	0.51	120	80	10.9	0.51	227.2	0.430	22718
	0.59	120	80	6.9	0.59	52.2	0.669	5223
	0.86	90	60	6.1	0.86	0.0	0.337	0
	0.70	100	70	10.3	0.70	1.5	1.407	152
	0.89	100	70	7.5	0.89	1.5	0.662	152
	1.19	150	80	8.9	1.19	0.0	1.327	0
	0.50	110	70	8.9	0.50	11.7	1.691	1166
	0.77	130	90	9.8	0.77	0.0	0.714	0
	0.45	120	70	6.7	0.45	0.0	0.485	0
	0.85	120	70	7.8	0.85	19.3	1.083	1927
	0.92	110	70	7.7	0.92	0.0	1.660	0
	0.42	140	90	9.2	0.42	1.5	0.516	152
	2.08	130	80	11.4	2.08	11.7	0.760	1166
	0.36	110	80	11.0	0.36	29.4	0.358	2941
	1.96	150	90	8.4	1.96	0.0	0.710	0
	0.56	110	80	8.4	0.56	11.7	1.566	1166
	0.61	140	80	9.6	0.61	34.5	1.236	3448
	0.90	140	80	6.5	0.90	42.1	1.242	4209
	0.55	140	90	8.6	0.55	1.5	0.668	152

	0.62	100	80	9.6	0.62	24.3	1.812	2434
	1.08	140	90	6.8	1.08	0.0	1.924	0
	0.68	120	80	6.5	0.68	37.0	3.465	3702
	0.52	140	90	7.2	0.52	0.0	1.104	0
	1.34	120	70	6.7	1.34	105.5	0.786	10548
	0.84	120	70	13.2	0.84	0.0	0.017	0
	0.50	100	60	13.1	0.50	19.3	0.385	1927
	0.36	140	80	9.0	0.36	156.2	1.960	15619
	1.25	120	80	8.8	1.25	0.0	1.162	0
	0.47	120	80	7.9	0.47	0.0	0.442	0
	0.57	120	80	8.2	0.57	24.3	1.259	2434
	0.79	110	80	6.3	0.79	19.3	1.304	1927
	1.84	160	80	7.4	1.84	0.0	0.788	0
	0.67	120	70	10.9	0.67	19.3	2.255	1927
	0.43	130	80	9.3	0.43	11.7	0.766	1166
	1.46	130	70	8.1	1.46	6.6	1.787	659
	0.89	110	70	8.2	0.89	236.0	2.572	23596
	0.93	120	80	9.4	0.93	0.0	0.997	0
	0.67	150	90	9.4	0.67	29.1	0.994	2911
	0.70	130	80	9.7	0.70	0.0	0.790	0
	0.54	140	90	9.0	0.54	0.0	0.332	0
	0.70	160	90	9.6	0.70	0.0	0.875	0
	0.49	120	80	10.7	0.49	133.8	0.609	13381
	0.68	110	80	6.9	0.68	0.0	0.795	0
	0.78	110	80	9.3	0.78	0.0	1.182	0
	1.55	140	90	8.8	1.55	18.9	1.396	1890
	0.63	130	70	11.5	0.63	59.8	0.044	5976
	1.33	120	80	7.9	1.33	0.0	1.090	0
	0.55	130	80	11.7	0.55	36.8	1.215	3677
	0.66	130	80	9.5	0.66	0.0	0.786	0
	1.24	120	80	6.4	1.24	39.3	1.741	3933
	0.71	130	80	8.5	0.71	57.2	1.175	5720
	0.68	120	70	8.1	0.68	0.0	1.062	0
	0.57	110	70	11.7	0.57	0.0	0.564	0
	0.42	130	90	6.6	0.42	0.0	0.167	0
	1.57	130	80	7.5	1.57	0.0	3.229	0
	0.70	110	70	11.0	0.70	884.6	0.571	88458
	0.63	140	90	10.9	0.63	0.0	0.302	0
	0.77	120	80	8.3	0.77	164.5	0.111	16445
	0.73	110	80	12.5	0.73	103.2	0.337	10317
	1.88	100	70	9.5	1.88	0.0	1.160	0
	0.51	120	80	11.4	0.51	18.9	0.682	1890
	0.67	120	80	8.6	0.67	0.0	0.856	0
	0.47	120	90	8.8	0.47	0.0	0.259	0
	0.47	140	70	9.2	0.47	0.0	0.985	0
	0.90	120	80	8.1	0.90	0.0	0.609	0
	0.68	110	80	7.9	0.68	0.0	0.075	0
	1.02	140	70	8.9	1.02	0.0	0.807	0

	0.76	120	70	10.0	0.76	0.0	3.388	0
	0.92	120	80	8.8	0.92	72.5	1.604	7252
	1.50	160	90	10.5	1.50	44.4	0.865	4443
	0.70	150	90	9.2	0.70	1837.1	1.193	183708
	0.50	120	70	9.9	0.50	0.0	1.541	0
	0.71	140	80	9.0	0.71	0.0	1.040	0
	0.42	120	80	6.4	0.42	0.0	1.464	0
	0.53	100	70	11.9	0.53	24.0	0.752	2400
	1.48	100	70	8.4	1.48	0.0	0.442	0
	0.65	140	80	10.7	0.65	223.2	0.524	22319
	0.72	130	80	8.9	0.72	6.1	0.013	613
	0.30	120	80	11.3	0.30	0.0	1.523	0
	0.69	130	70	8.1	0.69	11.2	0.685	1124
	0.44	150	80	9.4	0.44	192.5	0.152	19254
	0.58	120	80	7.9	0.58	0.0	1.166	0
	0.58	130	80	8.1	0.58	0.0	0.154	0
	0.56	130	90	7.5	0.56	0.0	0.407	0
	0.95	130	70	7.3	0.95	0.0	0.892	0
	0.52	130	80	8.4	0.52	172.1	0.281	17211
	0.95	190	90	11.1	0.95	0.0	0.319	0
	1.11	110	70	6.3	1.11	861.6	0.568	86159
	0.73	110	60	12.8	0.73	205.3	0.740	20531
	0.89	140	80	9.1	0.89	110.8	0.968	11083
	1.69	140	70	5.6	1.69	1.0	0.567	102
	0.54	130	80	10.0	0.54	0.0	0.444	0
	1.21	140	70	9.0	1.21	1.0	1.209	102
	0.36	110	70	12.3	0.36	0.0	1.517	0
	1.05	130	90	10.2	1.05	29.1	1.321	2911
	3.71	140	80	7.0	3.71	67.4	1.824	6742
	0.42	120	80	12.1	0.42	0.0	1.216	0
	0.77	100	70	8.2	0.77	187.4	0.444	18744
	1.00	110	70	7.2	1.00	34.2	0.334	3422
2		110	70	8.6	0.62	52.0	0.498	5200
7		130	80	11.6	1.00	8.8	0.746	880
2		110	70	9.6	0.62	5.0	0.294	500
3		110	70	7.9	0.62	33.5	0.927	3350
1		110	70	6.4	0.59	9.6	0.339	960
8		100	70	8.4	0.52	5.0	0.464	500
8		110	80	7.6	0.51	200.0	2.036	20000
7		110	70	8.1	0.78	196.0	0.407	19600
		130	80	8.2	0.63	50.2	1.832	5020
10		130	80	12.3	0.47	7.3	0.713	730
3		110	70	9.4	0.77	5.0	0.758	500
4		150	90	8.5	0.56	17.0	0.373	1700
7		100	70	7.0	0.69	5.0	0.837	500
1		110	70	6.9	0.54	5.0	0.407	500
24		110	70	12.1	0.75	28.4	2.624	2840
5		120	80	9.9	0.62	196.0	0.407	19600

10		130	80	9.2	0.53	18.8	1.911	1880
2		120	80	6.6	0.72	7.6	0.837	760
1		120	80	7.0	0.57	8.0	0.577	800
9		120	80	8.6	0.70	17.6	1.086	1760
		130	80	10.1	0.69	31.4	2.036	3140
5		120	80	8.9	0.81		0.837	0
		110	70	8.6	0.68	10.8	1.086	1080
19		110	70	8.2	0.73	125.3	0.486	12530
		120	80	6.5	0.45	200.0	1.730	20000
15		120	80	9.3	0.68	74.2	1.719	7420
		100	70	7.6	0.75	200.0	0.317	20000
19		130	80	8.6	1.10	10.1	0.385	1010
		130	80	8.9	1.16	200.0	0.430	20000
		100	70	9.9	0.76	50.9	0.995	5090
18		140	90	10.7	0.71	5.1	0.260	510
		130	80	9.2	0.77	200.0	1.595	20000
		130	80	6.5	0.77	98.8	0.927	9880
		110	70	7.8	0.83	5.0	0.215	500
		130	80	11.9	0.77	73.6	1.990	7360
		130	80	7.9	0.94	70.3	0.611	7030
12		130	80	6.1	0.89	5.0	0.565	500
9		130	80	6.0	0.88	5.0	0.622	500
5		100	70	8.7	0.79	8.4	0.667	840
6		120	70	9.2	0.78	5.0	0.317	500
19		120	70	10.0	0.94	8.9	0.351	890
5		100	60	8.2	1.00	200.0	0.667	20000
		130	80	7.6	0.86	62.3	0.554	6230
10		130	80	6.3	0.73	6.4	0.893	640
7		130	80	10.5	0.92	5.0	0.554	500
		130	80	8.0	0.76	10.3	1.063	1030
10		130	80	6.9	0.71	36.2	0.407	3620
6		100	70	9.1	0.74	142.1	1.459	14210
15		120	80	11.5	0.90	88.0	0.871	8800
4		140	80	9.2	1.05	200.0	0.837	20000
		110	70	6.1	1.09	5.3	1.086	530
		130	70	9.8	0.96	5.0	0.305	500
		130	80	6.9	0.77	0.0	0.837	0
10		110	70	7.1	1.12	5.0	1.108	500

Urin kreatinin (mg/dl)	Exercise Routine	Smoking habit
51.4		
26.5		
17.9		
71.9		
79.9		
97.7		
26.8		
134.5		
184.5		
49.1		
45.2		
17.4		
121.9		
95.4		
25.5		
87.1		
83.4		
66.1		
52		
50.2		
28.2		
85.2		
123.8		
41.1		
84.8		
69		
107.9		
127.1		
61.9		
41.5		
62.6		
63.7		
137.3		
43		
92.3		
23.1		
2.9		
89.1		
18.5		
116		
99.5		
103.5		
126.8		
31.5		
123.3		

Afinion ACR

EU unit	The Afinion ACR reportable ranges are the following: **5.0 t**
range urin albumin	5.0 to 200.0 mg/L for albumin
range urin kreatinin	1.5 to 30.0 mM for creatinine
range UACR	0.1 to 140 mg/mmol
US unit	
range urin albumin	16.4 to 416,3877.5 mcg/dl
range urin kreatinin	16.4 to 339.9 mg/dL
range UACR	1.0 to 1225.0 mg/g atau mcg/mg

100		
47.7		
3		
30.7		
46.1		
20.6		
21.4		
31.7		
92.1		
27.2		
72		
37.3		
45.1		
83.6		
15.3		
128.1	0	0
56.3	1	0
60.3	0	0
219.3	1	0
68.1	1	1
66.5	1	0
59.8	1	0
69.2	1	0
171.2	0	0
143.1	1	0
10.5	0	0
16.6	1	0
104.4	1	0
9.2	0	0
43	0	0
66.9	1	0
33.7	1	0
140.7	0	0
66.2	1	0
132.7	0	0
169.1	1	0
71.4	0	0
48.5	0	0
108.3	1	0
166	1	0
51.6	1	0
76	1	0
35.8	1	0
71	1	0
156.6	1	0
123.6	1	0
124.2	1	0
66.8	1	0

181.2	1	0
192.4	1	0
346.5	0	1
110.4	1	0
78.6	1	0
1.7	0	0
38.5	1	0
196	1	0
116.2	0	0
44.2	0	0
125.9	0	0
130.4	1	0
78.8	0	0
225.5	0	0
76.6	0	0
178.7	1	0
257.2	1	0
99.7	1	0
99.4	1	0
79	1	0
33.2	1	0
87.5	0	0
60.9	1	0
79.5	1	0
118.2	0	0
139.6	0	0
4.4	1	0
109	1	0
121.5	1	0
78.6	1	0
174.1	0	0
117.5	1	0
106.2	1	0
56.4	1	0
16.7	0	0
322.9	1	0
57.1	1	0
30.2	0	0
11.1	1	0
33.7	0	0
116	1	0
68.2	1	0
85.6	1	1
25.9	1	0
98.5	1	0
60.9	1	0
7.5	1	0
80.7	0	0

338.8	1	0
160.4	1	0
86.5	0	0
119.3	1	0
154.1	1	0
104	0	0
146.4	1	0
75.2	1	0
44.2	1	0
52.4	0	0
1.3	1	0
152.3	1	0
68.5	1	0
15.2	0	0
116.6	0	0
15.4	1	0
40.7	1	0
89.2	1	0
28.1	1	0
31.9	1	0
56.8	1	0
74	1	0
96.8	1	0
56.7	1	0
44.4	0	0
120.9	0	0
151.7	1	0
132.1	0	0
182.4	1	0
121.6	1	0
44.4	1	0
33.4	1	0
49.8	0	0
74.6	1	0
29.4	1	0
92.7	0	0
33.9	1	0
46.4	0	0
203.6	0	0
40.7	1	0
183.2	1	0
71.3	0	0
75.8	0	0
37.3	1	0
83.7	1	0
40.7	1	0
262.4	1	0
40.7	1	0

191.1	1	0
83.7	0	0
57.7	0	0
108.6	1	0
203.6	0	0
83.7	0	0
108.6	1	0
48.6	0	0
173	0	0
171.9	1	0
31.7	0	0
38.5	0	0
43	0	1
99.5	1	0
26	0	0
159.5	0	0
92.7	0	0
21.5	1	0
199	1	0
61.1	1	0
56.5	0	0
62.2	0	0
66.7	1	0
31.7	0	0
35.1	1	0
66.7	0	0
55.4		0
89.3	0	0
55.4	0	0
106.3	0	0
40.7	0	0
145.9	1	0
87.1	1	0
83.7	0	0
108.6	0	0
30.5	1	0
83.7	1	0
110.8	1	0

o 200.0 mg/L for albumin, 1.5 to 30.0 mM for creatinine (EU units), and 0.1 to 140 mg/mmol for ACR (European Union). The corre

esponding United States units for creatinine and ACR are 16.4 to 339.9 mg/dL and 1.0 to 1225.0 mg/g, respectively.
